# Annotations

**Published:** 1911-05-13

**Authors:** 


### Annotations.

### The Medical Treatment of London School Children

The recent meeting of medical men in the Marylebone district, convened to discuss the treatment of physically defective school children, has resulted in the adoption of a series of recommendations which need careful consideration by all interested in the subject. We have always insisted in these columns that the proper method, in our opinion, to deal with the question of such treatment is by the establishment of effective school clinics, and we are glad to find that the main trend of the resolutions is in the same direction. The present system of hospital tinkering, especially as it is done at hospitals with medical schools, is disgraceful alike to the institutions and to the profession, and it is time for the London County Council to grasp the significance of the facts and to establish school clinics, at least in one or two particularly badly served districts on the south side of the river.

### Measles and German Measles.

For some months now the inquest columns in the lay press have borne testimony to the high fatality rate of measles. Coroner after coroner has done his best to leaven the lump of maternal ignorance and carelessness by impressing on his juries and his reporters the gravity of this disease, still so universally regarded as trivial. In one way and another knowledge will, one supposes, gradually supplant tradition and prejudice among the proletariat on this subject; at present, apparently, almost the whole population still regards measles as a thing to be encouraged in its offspring, on the ground that the latter are sure to acquire it some day, and may just as well have it at once and be done with it. Nor, indeed, is it only among the poor and uneducated that this mischievous fallacy circulates; it still claims many adherents among those who ought to know better. It cannot be too widely made known that measles is a disease which no child need have or ought to have; and that it is one of the most dangerous of the infectious fevers of childhood, especially in its after-results. Just at present it seems as though measles itself has been supplanted a good deal by that mysterious and mild disorder *rötheln*, so unfortunately known as German measles. It is to be hoped that some day the ætiology of all these exanthematous fevers will be worked out properly; when that day arrives it may provide a solution of the problems of this curious disease, so extremely contagious and more prone perhaps than any other to occur in occasional widespread epidemics. If this disease were as dangerous as true measles, it would be a serious matter; for at present it seems to be raging all over the country. Notwithstanding this fact, it is still to be remembered that by the minutest care and by thoroughly intelligent isolation from the outset of contacts and suspects, German measles can be checked when it invades a large community of young and susceptible persons, such as a school. Since the present outbreak began there have been several notable instances of this, some of which have been published. It behoves every parent and guardian to take expert advice whenever the disease breaks out in those under their charge, for only thus can the spread of these extensive epidemics be prevented.

### A Fine for Celibates.

Senator Beall, of Alton, has just presented to the Senate of Illinois his Bill for the encouragement of fecundity and the prevention of celibacy. In consideration of the fact that despite the paternal dispositions of the Government the population of the State increases even less quickly than that of France and is composed of at least a third unproductive boys and girls, Mr. Beall's Bill ordains that every celibate over thirty-five years of age who cannot produce a satisfactory reason for his solitary condition shall have to pay a yearly tax of £2. The product of this tax will be devoted to the population fund created by the same Bill and intended to recompense prolific parents. Mothers will be rewarded with a gratuity of £20 for each child born after two years of married life. It was probably thought that the firstborn needed less encouragement. Twins will give the right to double, and triplets to three times the gratuity.

### A Remarkable Malapraxis Suit.

Surely the most extraordinary action ever brought for alleged *malapraxis* must be that which has recently been successfully defended by Dr. Smith, of Los Angeles, in the United States. Put very briefly, this surgeon operated upon a patient, discovered a growth which he decided could not safely be removed, and performed a colostomy to relieve the symptoms of obstruction. The patient was dissatisfied with the result, and indeed in many cases the condition of a man with a colostomy opening is pitiable. Half a dozen eminent medical witnesses testified to their faith in the diagnosis of a tumour; but two were produced by the defence who said they did not believe in its existence. The plaintiff had such faith in these two, and was so exasperated against his former surgeon, that he submitted to a further exploratory operation to settle the point! The tumour was indisputably proved to exist, and the patient sacrificed his life, as it turned out, in disproving his own case. The trial occupied nine days, and the sum claimed as damages was fifty thousand dollars; the jury found a verdict for the defendant after the briefest of retirements. One of the few gratifying features about the whole stupid business is the fact that the new Medical Defence Society of the State of California thus won its first action at law in defence of a member. This inaugural success, especially in a case of such magnitude and interest, will inevitably do a great deal of good in more ways than one. It must force home to the medical profession in California the immense value of the organisation and it will enhance the prestige of the latter in such a way that speculative and blackmailing actions brought against it are likely to be discouraged most effectively.

